# Biomedical event trigger detection by dependency-based word embedding

**DOI:** 10.1186/s12920-016-0203-8

**Published:** 2016-08-10

**Authors:** Jian Wang, Jianhai Zhang, Yuan An, Hongfei Lin, Zhihao Yang, Yijia Zhang, Yuanyuan Sun

**Affiliations:** 1 School of Computer Science and Technology, Dalian University of Technology, Dalian, China; 2 College of Computing & Informatics, Drexel University, Philadelphia, USA

**Keywords:** Biomedical event extraction, Trigger detection, Dependency-based word embedding, Neural network

## Abstract

**Background:**

In biomedical research, events revealing complex relations between entities play an important role. Biomedical event trigger identification has become a research hotspot since its important role in biomedical event extraction. Traditional machine learning methods, such as support vector machines (SVM) and maxent classifiers, which aim to manually design powerful features fed to the classifiers, depend on the understanding of the specific task and cannot generalize to the new domain or new examples.

**Methods:**

In this paper, we propose an approach which utilizes neural network model based on dependency-based word embedding to automatically learn significant features from raw input for trigger classification. First, we employ Word2vecf, the modified version of Word2vec, to learn word embedding with rich semantic and functional information based on dependency relation tree. Then neural network architecture is used to learn more significant feature representation based on raw dependency-based word embedding. Meanwhile, we dynamically adjust the embedding while training for adapting to the trigger classification task. Finally, softmax classifier labels the examples by specific trigger class using the features learned by the model.

**Results:**

The experimental results show that our approach achieves a micro-averaging F1 score of 78.27 and a macro-averaging F1 score of 76.94 % in significant trigger classes, and performs better than baseline methods. In addition, we can achieve the semantic distributed representation of every trigger word.

## Background

With the development of system biology which emphasizes the importance of relations and interactions between biological entities, revealing biomedical events, the complex interactions between biological molecules, cells, and tissues, becomes imperative [1]. Biomedical events play a key role in the development of biomedical research, which can contribute to biomedical database development and pathway curation. However, there is little knowledge about biomedical events in existing databases that we can utilize directly. Consequently, with the rapidly growing quantity of biomedical scientific literature, continuing effort must be put into mining the underlying knowledge (e.g. entity relations and biomedical events) hiding in the scattered literature. As such, biomedical event extraction has attracted much attention. BioNLP shared tasks [2] have been held for three editions aiming to extract fine-grained, complex, and structural events from biomedical scientific literature and many novel methods have been proposed.

**Fig. 1:**
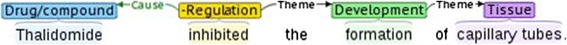
The annotation example of biomedical event

In general, we define an event as a triple tuple: <event type, theme, cause>. The event type denotes the related event behavior, such as gene expression, regulation, or binding. An event contains the theme and cause, where the theme is the primary participant of the event and the cause is the reason that the event behaves. For instance, as shown in Fig. 1, the words “inhibited” and “formation” denote the events “Regulation” and “Development,” respectively. We call the event “inhibited” a complex event that contains another event and the event “formation” a simple event that only contains the theme. No matter is an event simple or complex, the trigger plays a major role in the whole event extraction procedure.

The current popular approaches for biomedical event extraction mainly follow a pipeline procedure: event trigger detection and event argument extraction. In such pipeline methods, event trigger detection plays a primary role. Our preliminary analysis has shown that more than 60 % of extraction errors are caused by trigger detection. Unsurprisingly, much effort [3, 4] has been put into improving the performance of trigger detection.

There are mainly two kinds of approaches for trigger detection: rule-based and machine learning approaches. Rule-based approaches focus on the definition of a set of extraction rules, such as regular expression and matching pattern rules. However, it is much difficult to define different rules to match all trigger words, which influences the overall performance of trigger detection. Furthermore, rule-based methods fail to generalize to the new dataset, which is an unavoidable problem in extracting new events between new biological entities.

Machine learning approaches treat the trigger detection task as a traditional classification problem that assigns an event label to every word. These kinds of methods usually extract high-end hand-designed features from processed training data. For training a trigger detection model [5], the features are fed into a classification model, such as Support Vector Machine (SVM). However, the annotated data we can utilize is often not enough for training a classification model with acceptable performance. Consequently, Zhou [6] proposed a novel method that learns domain knowledge from a large corpus of text and embeds it into word features with a natural language model. In these approaches, the hand-designed features generally reach hundreds of thousands of dimensions in order to better represent the trigger feature information that will be fed into the classification algorithm. To obtain this useful classification information, the method usually needs to parse the data using a shallow and deep dependency parser which is usually time and computation consuming. Afterwards, features are manually designed through the parse results. However, these kinds of methods need to design different features for different NLP tasks based on the particularities of each specific task. Furthermore, the hand-designed features are traditional one-hot features lacking semantic information about the trigger. Nowadays, there are many methods for modeling words’ semantic information. Of these, word2vec [7] is one of the most popular tools because of its effectiveness and efficiency.

We propose a biomedical event trigger detection method by employing neural network model and dependency-based word embedding for addressing the complex problem of manually designing task-specific features. The method aims to not only solve the problem of dimension disaster but also can be generalized to new extraction tasks with new data without extra intervention. The dependency-based word embedding is learned from all available PubMed abstracts which have similar topic with the annotated data. The embedding contains more functional semantic information. Words have higher similarity when they behave similar in function. Consequently, the dependency-based word embedding contributes more to the trigger detection task than topical word embedding does since the classification of trigger needs more functional information. Then the senior and significant features are automatically learned by the neural network model (also called deep learning model). For evaluating the effectiveness of our proposed approach, we perform the experiment in MLEE dataset to extract the biomedical event trigger containing 19 trigger classes. Our experimental results show that the method has better performance compared to baseline approaches.

The proposed method mainly contains following contributions: (1) Dependency-based word embedding is employed to address the functional semantic information in the trigger detection task. (2) Features are automatically built from raw input with dependency-based word embedding. (3) Neural network model is utilized to learn powerful features which are fed to softmax classifier. Meanwhile, the word embedding is dynamically adjusted based on backup propagation algorithm.

The remaining part of the paper is organized as follows. Section II detailed illustrates the proposed method. Section III presents the experimental results and detailed discussion. Section IV concludes our paper and describes the future work.

## Method

**Fig. 2:**
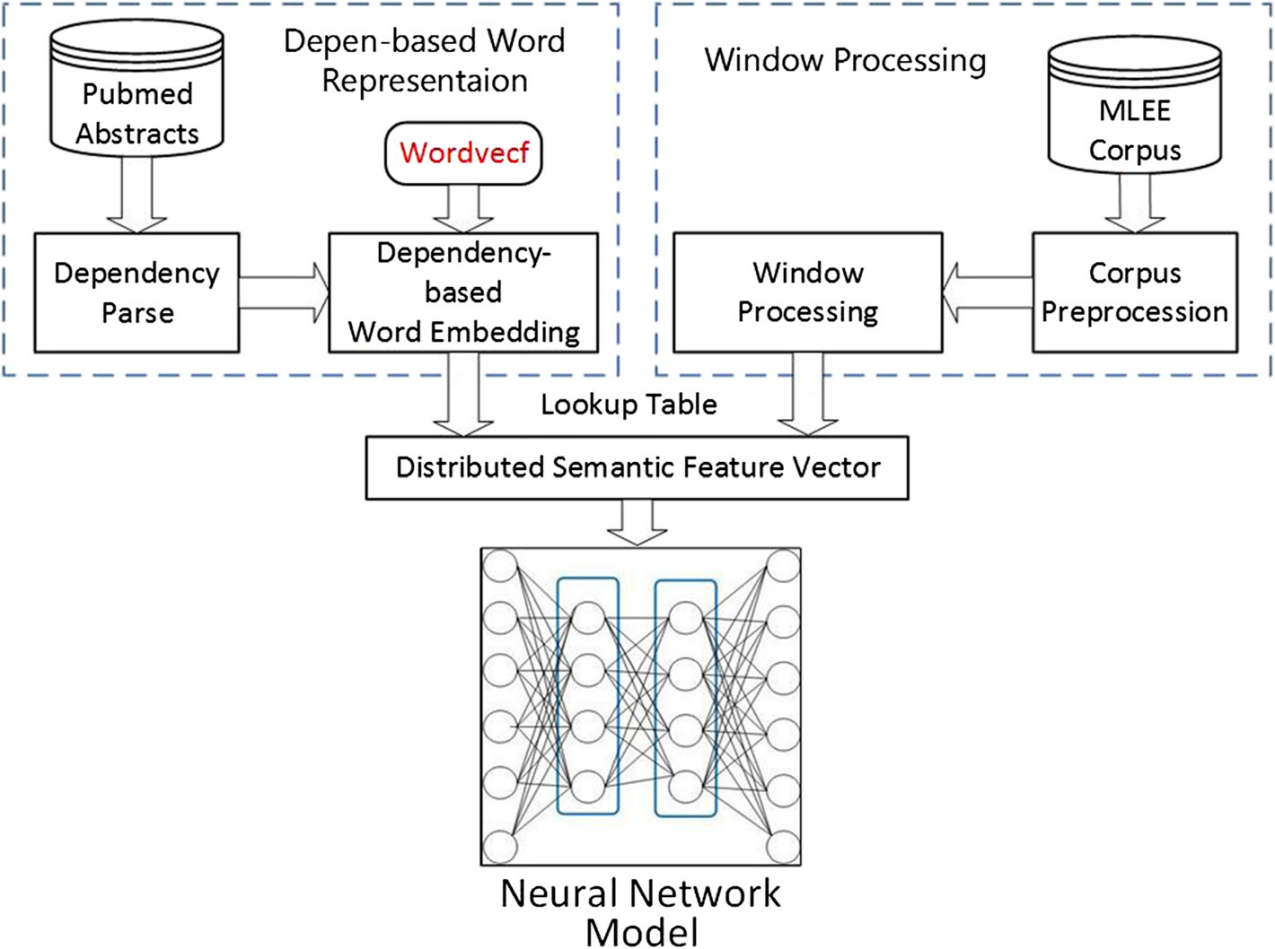
The framework of our method

Our method consists of four major parts as shown in Fig. 2: (1) Dependency-based word embedding is trained by Wordvecf [8] with all available PubMed abstracts. The embedding contains more functional information, which contributes more to our trigger identification task in that most of the detected triggers are verbs or words acting as verbs. (2) The distributed semantic feature vectors, which have low dimension and continuous value, are extracted from the processed dataset by a simply window-based approach without extra preprocess. (3) The neural network classification model is employed to automatically learn the hidden and significant feature representation from the raw input and train the trigger detection system with the labeled dataset based on a back propagation algorithm. At same time, the algorithm dynamically adjusts the input word embedding to learn the better word embedding for the trigger detection task. (4) The biomedical event triggers are predicted by the softmax layer.

### Biomedical knowledge

The annotated dataset only contains 262 documents with annotations. We cannot learn the exact semantic information from such little data. However, there are large amounts of biomedical knowledge hiding in public databases. Consequently, we employ the public available biomedical knowledge, PubMed abstracts, to accurately capture the semantic information of every word, especially event trigger words to promote the performance of trigger identification. The data are approximately 20G, which are enough to model the semantic information of every word.

### Word embedding

The machine learning algorithm requires some fix-length feature vectors, such as bag-of-words [9]. Despite its popularity, the bag-of-words lacks the order information and semantics. So, word embedding, also called word vector or word distributed representation, aims to present the semantics of words and lower the feature dimension with a low-dimension semantic vector instead of a high dimensional and sparse feature vector. Specially, we can learn different word embedding for different tasks. Of all word embedding, word2vec [7] achieves giant success in many NLP tasks, such as Named Entity Recognition(NER) and Part-of-Speech(POS) Tagging. There are mainly two kinds of word embedding: bag-of-words-based embedding and dependency-based embedding.

1. Bow-based word embedding
  The linear bag-of-words contexts are employed to train word embedding by word2vec and many other neural language models. The method uses a window of size k to predict the current word or current word to predict its surrounding words. Generally, we utilize the skip-gram model to maximize the average log probability:

1 $$ \frac{1}{T}{\displaystyle \sum_{t=1}^T}{\displaystyle \sum_{-c\le j\le c,j\ne 0}} \log \wp \left({w}_{t+j}\Big|{w}_t\right) $$

where c is the window size (a hyper-parameter to be chosen while training). We call this kind of word embedding bow-based word embedding, which is trained by the linear contexts. The embedding is full of semantic information, usually topic semantics. For example, the word “dancing” is most similar to the words “dance,” “dances,” and “dancers.”

**Fig. 3:**
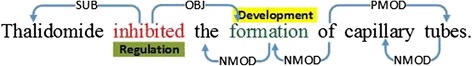
Example of a dependency parse result

2) Dependency-based word embedding
  Unlike other NLP tasks, such as POS or CHUNCK, the trigger detection task needs more information in dependency contexts than in linear contexts. Consequently, an alternative to the bag-of-words approach is to derive contexts based on deep dependency parse. As shown in Fig. 3 and Table 1, after parsing each sentence, we derive word contexts in syntactic relations and use them to train word embedding. And we can capture relations of words that are far apart and thus “out-of-reach” with small window bag-of-word.

**Table 1:** Dependency Contexts

Words	Dependency contexts
Thalidomide	inhibited/SUB
Inhibited	Thalidomide/SUB-1^a^,formation/OBJ
The	formation/NMOD-1^a^
Formation	the/NMOD, of/NMOD-1
Of	activity/NMOD, tubes/PMOD-1^a^
Capillary	tubes/NMOD-1^a^
Tubes	capillary/NMOD, of/PMOD-1^a^

^a^’-1’ refers to the inverse relation

More specifically, we parse all available PubMed abstracts with Gdep parser [10], a dependency parse tool specialized for biomedical texts, and train the dependency-based word embedding based on the contexts in dependency relations with the tool word2vecf [8].

Generally, this kind of word embedding model derives more functional semantic information. For example, the word “dancing” is most similar to the words “singing,” “rapping,” and “miming,” which act in similar roles in a sentence. This kind of functional semantic information is the important resource for our trigger identification task.

### Corpus preprocessing

In most trigger recognition methods, complex preprocessing, such as shallow and deep parse, usually need to be made for extracting complex, high-end, and useful features that are fed to the specific classifier. However, in our method, there is no need to do such complex preprocessing. In fact, we do not even use entity annotation information, which takes significant time to annotate.

In our method, only the following steps are employed to process the annotated data.

- Sentence split. There are no events with cross sentences in the dataset. Consequently, we utilize the GENIA Sentence Splitter [11] to split the source text into the sequence of sentences for facilitating further processing.
- Tokenization. Tokenization refers to the process of breaking the input text into words, phrases, and symbols that become the input of further processing. We employ the NLTK toolkit [12], which is a widespread used natural language toolkit, to tokenize a sentence into the sequence of tokens.

### Word embedding based neural network model

**Fig. 4:**
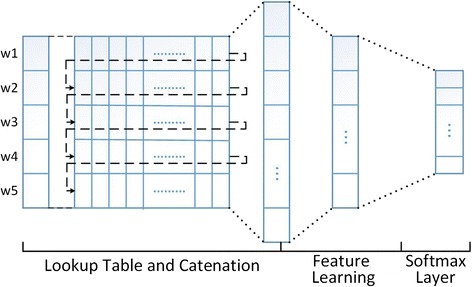
Neural network model

As described previously, the trigger detection task can be seen as a classification task. Traditional trigger identification methods mainly extract high-end, hand-designed features from the data with being processed by kinds of NLP methods containing sentence split, tokenization, and dependency parse. Then feature extraction is finished based on these parse results and the annotated entity information. This is a complex and task-dependent process. Consequently, it is difficult to generalize to new data or new task. Instead, we employ the dependency-based word embedding with rich functional semantic information to automatically learn significant features using a general purpose learning procedure (called deep learning [13]). Our methods follow the process as shown in Fig. 4.

### Window processing

Inspired by word2vec [7], we can predict the trigger word by its context information with rich semantic information that is called distributed semantic representation. Consequently, we only utilize the linear context of the target trigger to identify the target trigger, instead of extracting complex features from the dependency tree or entity annotation.

For example, as shown in Fig. 3, “inhibited” is a trigger with type “Regulation”. We use the words “Thalidomide” and “formation”, where the word “the” is a stop-word to predict the word “inhibited”, whether it is a trigger and which types it belongs to. Although the method looks simple, it is effective. As a result, in this step, we process every instance into the sequence of word indices, which are fed into the next steps.

### Lookup table layer

Then, the sequence of word indices are projected into the word vector through the lookup table layer and concatenating operation. The operation of lookup table refers to projecting every word in a specific window to the semantic representation which is utilized to predict the trigger. And the concatenating operation refers to joining every single vector of the word end to end into the corresponding feature vector. The vector is regarded as the semantic feature representation of the target trigger. Then, the generated vectors are fed into the neural network model to train the trigger detection model by a backup propagation and gradient descent algorithm.

More formally, for each word $\mathrm{w}\in \mathcal{D}$, the responding vector representation is acquired by the lookup table layer ${LT}_w\left(\bullet \right)$:

2 $$ L{T}_W\left(\mathrm{w}\right)={W}_w^1 $$

where $W\upepsilon {\mathbb{R}}^{\left|\mathcal{D}\right|\times size}$ is the word matrix parameter being learned using PubMed abstracts and fine-tuning the training process to be more adaptive to the specific dataset. ${\left\langle W\right\rangle}_w^1$ is the ${w}^{th}$ column of *W* and *size* is the word vector size (a hyper-parameter to be chosen) [14].

### Automatic feature learning

Conventional supervised machine learning (shallow classifier) methods are used to design a good feature extractor that requires a considerable amount of engineering skills and domain expertise, but the hand-designed feature doesn’t allow the learner to generalize well outside of training examples. In our methods, we employ the neural network model to automatically learn good feature representation from raw input for making the classifier more powerful. At each layer, the total input z is computed for each units which is the weighted sum of the outputs of the last layer. Then a nonlinear activation function $\mathrm{f}\left(\bullet \right)$ is applied to z to generate the output of the current unit. Among all nonlinear activation functions, the rectified linear unit (ReLU) $\mathrm{f}(z)= \max \left(0,x\right)$ is chosen, commonly used in recent years, for its effectiveness and fast convergence.

3 $$ \mathrm{H}(x)=f\left(\omega \cdot x+b\right) $$

Where *x* is the output of upper layer, $\omega$ is the parameter matrix between the layers, b is the bias item, and $\mathrm{H}(x)$ can be regarded as the senior feature representation, which can be classified better. Then the learned features are fed into the classifier to classify the sample into a specific trigger class.

### Training

After building the biomedical event trigger identification model, we need to choose an optimization algorithm to train the model and search for optimal parameters. Among most optimization algorithms, such as gradient descent and Newton method, gradient descent is the most efficient and popular algorithm because it is simple and requires less computation. Gradient descent is a first-order optimization algorithm to find the local minimum of a function. However, we usually employ batch gradient descent for saving expensive computation and its power of acquiring a better solution. We compute the partial derivative of objective function toward the parameter of each layer based on a backup propagation algorithm. Then we update the parameters of each layer using the gradient in that of fast convergence throwing the negative direction of gradient. The algorithm walks through all training examples with multiple iterations and stops iterating until the algorithm is convergence. Finally, we can acquire the optimal parameters of model and we can predict new examples using these optimal parameters.

In addition, we modify the neural network architecture with dropout architecture [15] and employ AdaDelta update rules [16] to update the parameters dynamically. The AdaDelta update rule dynamically adapts over time using only first order information and has minimal computational overhead beyond vanilla stochastic gradient descent. The dropout architecture refers to random dropout values of some nodes. This can prevent the complex co-adaptations in which a feature node can only be helpful in the context of several other feature nodes. Overfitting usually can be avoided by randomly omitting some feature nodes.

Finally, we implement the trigger identification model by Theano [17]. Theano is python library that allows users to define, optimize, and evaluate mathematical expressions. Consequently, we can implement the neural network model more easily. In addition, Theano supports convenient configuration for GPU running, which can greatly accelerate the training speed.

### Hyper-parameters

As described in the last section, we adopt AdaDelta update rule to update the parameters of the model. Consequently, we do not need to initialize the learning rate, which plays a considerably important role in the entire training process. This facilitates the process of choosing parameters to some extent. In addition to learning rate, there are some other hyper-parameters, such as word embedding size, dropout rate, layer number, layer size, and batch size. The different combinations of different hyper-parameters will lead to different results. As we all know, there are no methods to find the best combination of all the hyper-parameters theoretically. Consequently, we empirically search for the reasonable combination of all the hyper-parameters through a large number of experiments. The combination of hyper-parameters of the model is shown in Table 2.

**Table 2:** The combination of hyper-parameters of the model

Hyper-parameter	Layers	Word	Dropout	Batch
Value	4	200	0.5	256

## Experiment results and discussion

In order to evaluate the performance of our proposed biomedical trigger detection model, we conducted the experiment study with the MLEE dataset^1^, which aims to support event extraction across levels of biological organization from the molecular to the organ system level. At the same time, we also employ all the available PubMed abstracts to train the rich functional semantic information for every trigger word. Then we design a multi-layer neural network model based on dependency word embedding for trigger classification. The neural network architecture automatically learns the good features from raw word embedding without redundant processing and task dependence, and it classifies the event triggers by the learned hidden features. Furthermore, the raw word embedding is dynamically adjusted based on a backup propagation algorithm while training to be more adaptive to the specific dataset and represent more accurate functional semantic information of event triggers. At last, we compare our experiment results with other state-of-the-art methods based on precision, recall, and *F1* score.

### Dataset

The MLEE dataset mainly focuses on the topic about angiogenesis, a key process in tumor development. The dataset supports event extraction across more concrete entity and trigger types. The entities contains molecular, cell, tissue and organ and the related event triggers are divided into four categories containing 19 pre-defined trigger classes, such as “Regulation”, “Cell proliferation” and “Blood vessel development”. However, as shown in Table 3, there are distinct differences in trigger numbers among different trigger classes [18].

**Table 3:** The number of different trigger classes

Category	Event type	Number
	Cell proliferation	43
	Development	98
	Blood vessel development	305
Anatomical	Growth	56
	Death	36
	Breakdown	23
	Remodeling	10
	Synthesis	4
	Gene expression	132
	Transcription	7
Molecular	Catabolism	4
	Phosphorylation	3
	Dephosphorylation	1
	Localization	133
	Binding	56
General	Regulation	178
	Positive regulation	312
	Negative regulation	223
Planned	Planned process	175

### Evaluation metrics

As with most of classification tasks, we chose precision (*P*), recall (R), and F1 score (*F1*) to evaluate the performance of our trigger detection model for every trigger class.

4 $$ P=\frac{tp}{tp+fp} $$

5 $$ R=\frac{tp}{tp+fn} $$

6 $$ F1=\frac{2*P*R}{P+R} $$

Where *tp* is true positive for test examples, *fp* is false positive, and *fn* is false negative.

Furthermore, for evaluating overall performance, we employ the micro-averaging (7), and macro-averaging (8) methods to evaluate the overall *F1* score performance [19, 20].

7 $$ MacroAv{g}_{F1}=\frac{{\displaystyle {\sum}_{i=1}^{\left|C\right|}}F{1}_i}{\left|C\right|} $$

8 $$ MicroAv{g}_{F1}=\frac{{\displaystyle {\sum}_{i=1}^{\left|C\right|}}{\left(2*P*R\right)}_i}{{\displaystyle {\sum}_{i=1}^{\left|C\right|}}{\left(P+R\right)}_i} $$

Where *C* is trigger class, and $\left|C\right|$ is the responding class number.

As is the same of most of supervised machine learning methods, we cannot accurately predict the trigger whose instance number is rare. At the same time, the prediction failure of these triggers may have a serious impact on the overall performance. Consequently, we evaluate the overall performance ignoring the trigger class with rare quantities (less than 10).

### Performance analysis

For evaluating the efficiency of our proposed method, we compute the precision, recall, and *F1* score for each class and compare them with state-of-the-art methods. More specifically, we compare the results of dependency-based word embedding with bow-based word embedding and compare the results of non-static word embedding with static word embedding, which shows that our proposed method is efficient.

**Fig. 5:**
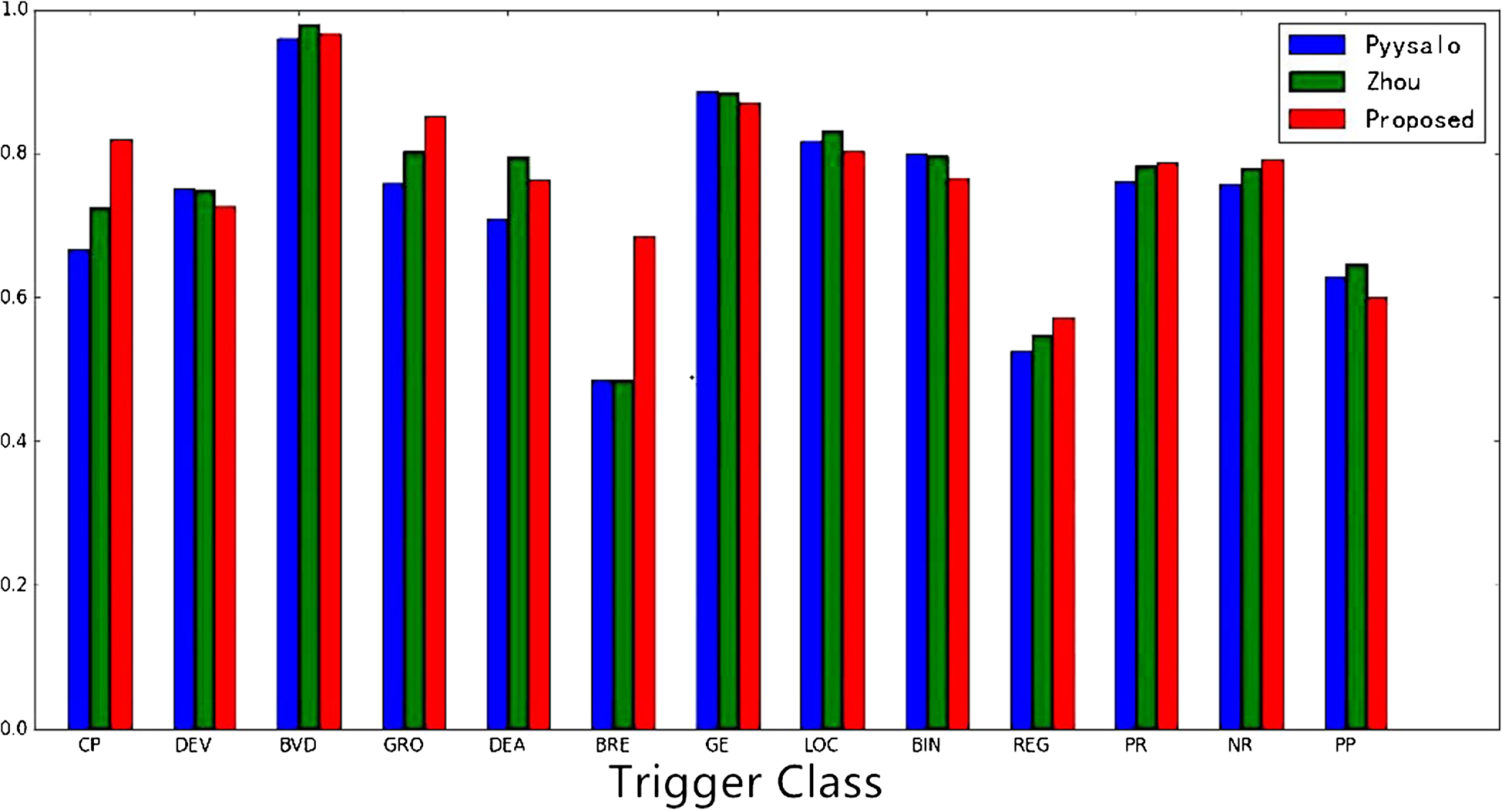
Experimental Results

1. Overall analysis and discussion
  We employ Pyysalo et al. [5] and Zhou et al. [6] as the baseline methods. Pyysalo et al. implemented an SVM-based approach, which manually designs salient features such as context and dependency features and fed them into SVM classifier. Zhou et al. also conducted a similar experiment. The method achieved significant results over existing methods. However, this method only utilizes the annotated data and fails to utilize the rich semantic information contained in massive amounts of biomedical literature. Zhou et al. employed a feedforward neural network to train word embedding and integrated it with hand-designed features into the SVM classifier. This method has achieved state-of-the-art results. However, as mentioned in [21], the feedforward neural network is not the optimal method for training word embedding compared with the Skip-gram model. Furthermore, this method still needs manually designed features, which limits the power of generalization.
  As shown in the Fig. 5, we compare our experimental results (only event types with more than 10) with Pyysalo et al. and Zhou et al. to show the potential of our proposed method. From Fig. 5, we can observe that there are eight classes that perform better than Pyysalo et al. and six classes that perform better than Zhou et al. As shown in Tables 4 and 5, it can be observed that we achieve better overall performance over micro-averaging and macro-averaging F1 score. More significantly, our proposed approach automatically learns significant feature representation based on dependency-based word embedding without any manual intervention and hand-designed features compared with the methods of Pyysalo et al. and Zhou et al. Consequently, our proposed approach has stronger power of generalization and it can be applied to new examples.

**Table 4:** Micro-averaging F1 score of significant events

Method	*R*(%)	*P*(%)	*F1 Score*(%)
Pyysalo [5]	81.44	69.48	74.99
Zhou [6]	80.60	74.23	77.28
Proposed	83.62	73.56	78.27

**Table 5:** Macro-averaging F1 score of significant events

Method	*R*(%)	*P*(%)	*F1 Score*(%)
Pyysalo [5]	78.04	68.74	73.09
Zhou [6]	79.18	72.03	75.43
Proposed	81.89	72.56	76.94
2. Dependency-based word embedding versus bow-based word embedding
  Most NLP tasks employ bow-based word embedding as semantic representation for its popularity and efficiency. However, it is not proper for the trigger detection task. In a trigger identification task, the target triggers usually are verbs or words acting as verbs. We cannot simply predict the trigger using bag-of-words because the target trigger is usually far away from entities, such as proteins and RNA. Consequently, we employ dependency-based word embedding for the trigger classification problem. Generally, dependency-based word embedding is trained based on syntax contexts instead of bag-of-words. More specifically, the syntax contexts are acquired by Gdep parsing. The dependency word embedding contains more functional semantic information.
  To verify the efficiency of dependency-based word embedding, we compare the experimental results of dependency-based word embedding and bow-based word embedding. As shown in Figs. 6 and 7. Figure 6 is the change of macro-averaging F1 score over iteration and Fig. 7 is the change of micro-averaging F1 score over iteration. From these two figures, it can be observed that dependency-based word embedding (“dep10” in Figs. 6 and 7 where “10” suggests that we filter the words less than 10) performs better than bow-based word embedding (“bow” in Figs. 6 and 7).

**Fig. 6:**
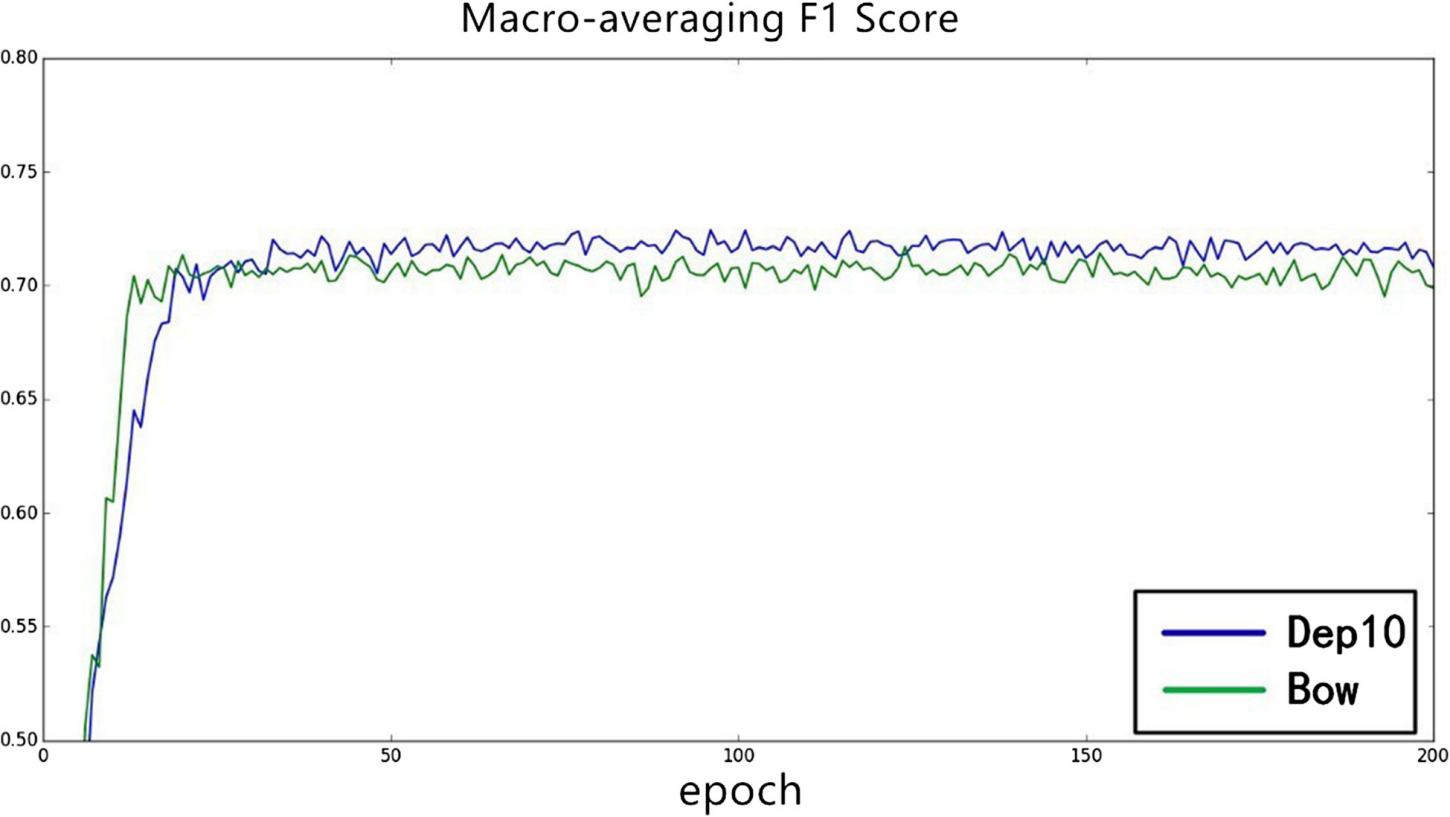
The influence of word embedding on Macro-averaging *F1* score

**Fig. 7:**
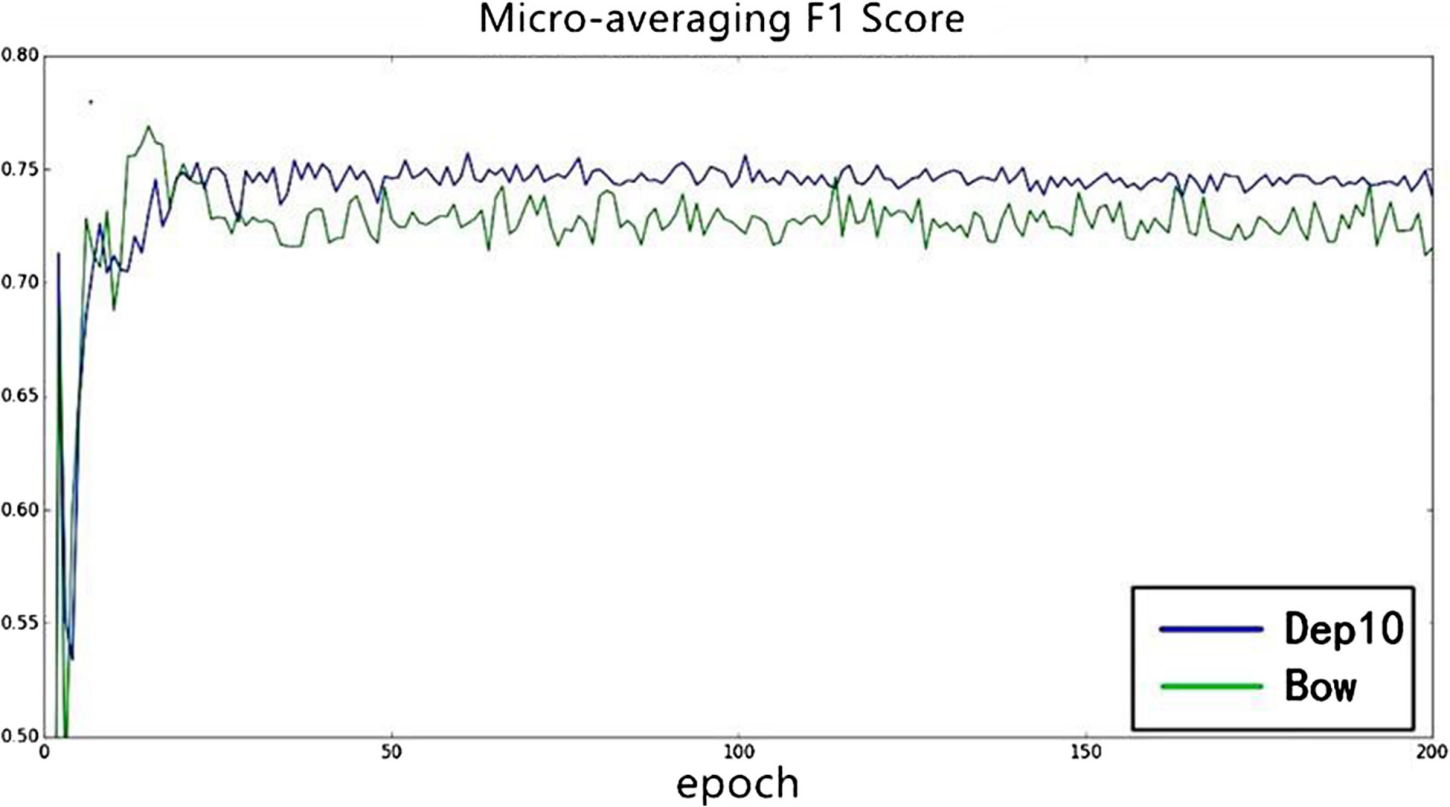
The influence of word embedding on Micro-averaging *F1* Score
  Through our experiments on different word embedding, we can conclude that different word embedding has a different influence on different tasks. Consequently, we must choose a specific word embedding (such as dependency-based word embedding in our task) based on different problems.
3. Word embedding static versus non-static
  In order to learn better trigger semantic information for our task in the specific dataset, we dynamically modify the trigger word embedding matrix based on a backup propagation algorithm and gradient descent algorithm while training. In this process, we adjust the word embedding parameters using the annotated data, which can be regarded as a supplement for unsupervised training.
  Different experiments are conducted to evaluate the different influence of static and non-static word embedding. Figure 8 is the changing of macro-averaging F1 score over iteration epochs, and Fig. 9 is the changing of micro-averaging F1 score. As shown in Figs. 8 and 9, word embedding with non-static achieves better experimental results (macro-averaging and micro-averaging F1 score) in stability and efficiency over iterations. Consequently, it is suggested that adjusting the word embedding in the training process not only makes the neural network model more stable and gives it stronger power of generalization but it can also achieve more optimal experimental results.

**Fig. 8:**
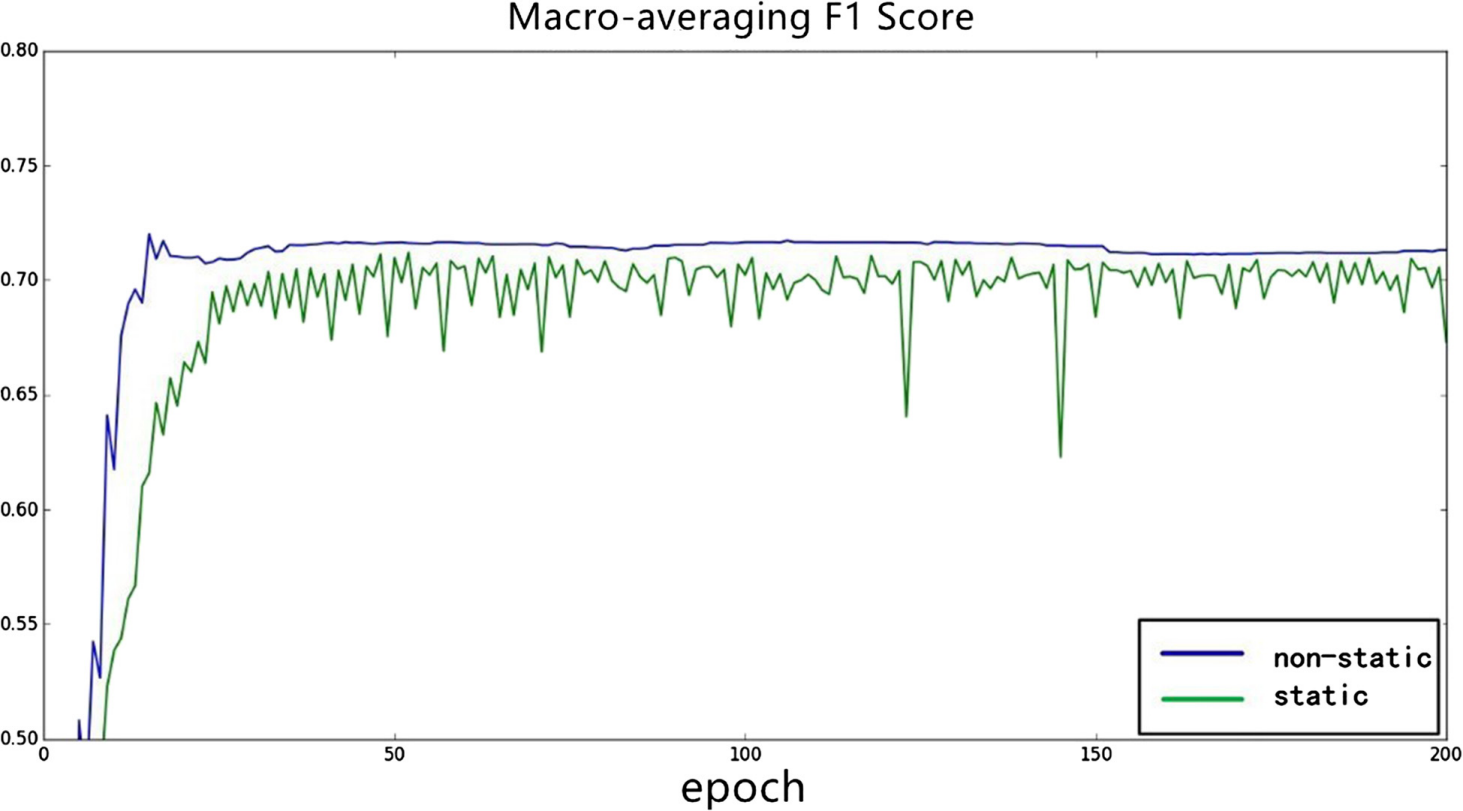
The influence of static and non–static on Macro-averaging *F1* score

**Fig. 9:**
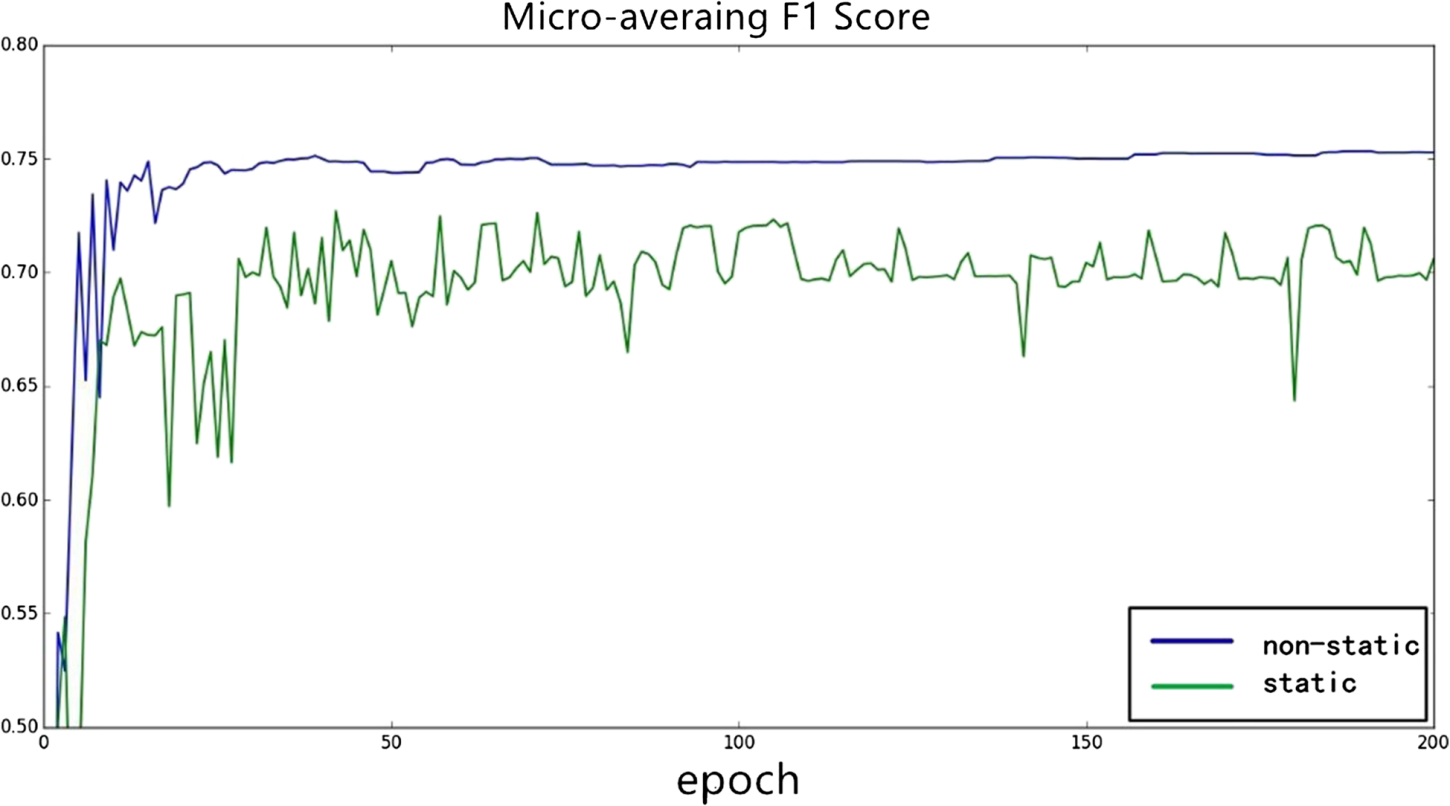
The influence of static and non-static on Micro-averaging *F1* score
  Finally, we can achieve semantic distributed representation (word embedding) for every trigger. The word embedding contains rich semantic information of the trigger.

## Conclusion and future work

In this paper, we proposed a method to automatically extract biomedical event triggers from biomedical texts. This method combines word embedding and neural network classification models to build the trigger identification architecture. The method takes advantage of word embedding with massive biomedical resources containing rich semantic information and does not need annotated information of the entity, which can save the expensive cost of annotating data. At the same time, we utilize distributed semantic vector instead of convolutional hand-designed features for its stronger power of generalization.

In addition, we employ dependency-based word embedding, which contains more functional semantic information, to better capture semantics of triggers. And we dynamically adjust word embedding based on supervised training. The experimental results show that our proposed approach is efficient compared with baseline methods and it can generalize better.

In the future, we will explore tree-based deep learning model such as tree LSTM which can automatically learn features from dependency tree for trigger detection task. And we will extract complete biomedical events after detecting triggers.

## Footnotes

1 http://nactem.ac.uk.
